# A Pollutant’s Tale: An Interactive Talk on the Chemistry of the Earth’s Climate and Its Response to Pollutants

**DOI:** 10.1021/acs.jchemed.4c00532

**Published:** 2025-04-03

**Authors:** Timothy G. Harrison, Michael T. Davies-Coleman, Alison C. Rivett, M. Anwar H. Khan, Joyce D. Sewry, Magdalena Wajrak, Nicholas M. Barker, Jonny Furze, Sophie D. Franklin, Linda Sellou, Naomi K. R. Shallcross, Dudley E. Shallcross

**Affiliations:** † School of Chemistry, Cantock’s Close, University of Bristol, Bristol BS8 1TS, U.K.; ‡ Department of Chemistry, University of the Western Cape, Robert Sobukwe Road, Bellville, 7535, South Africa; § Department of Chemistry, Rhodes University, Makhanda 6139, South Africa; ∥ School of Science, Edith Cowan University, 270 Joondalup Drive, Perth, WA 6027, Australia; ⊥ Social Inclusion Group, University of Warwick, Coventry CV4 7AL, U.K.; # Primary Science Teaching Trust, 12 Whiteladies Road, Bristol, BS8 1PD, U.K.; ○ Department of Chemistry, National University of Singapore, 3 Science Drive 3, Singapore 117543; △ Becket Primary School, Tavistock Rd, Worle, Weston-super-Mare BS22 6DH, U.K.

**Keywords:** **Audience**, General Public, Elementary, Middle School Science, High School, Introductory Chemistry, First-Year Undergraduate, General, Second-Year Undergraduate, Upper-Division Undergraduate, Graduate Education, Research: Continuing Education, **Domain**, Demonstrations, Environmental Chemistry, Interdisciplinary, **Pedagogy**, Multimedia based learning, **Topic**, **Acids/bases**, **alcohols**, **alkynes**, **catalysis**, **free radicals**, **gases**, **kinetics**, **photochemistry**

## Abstract

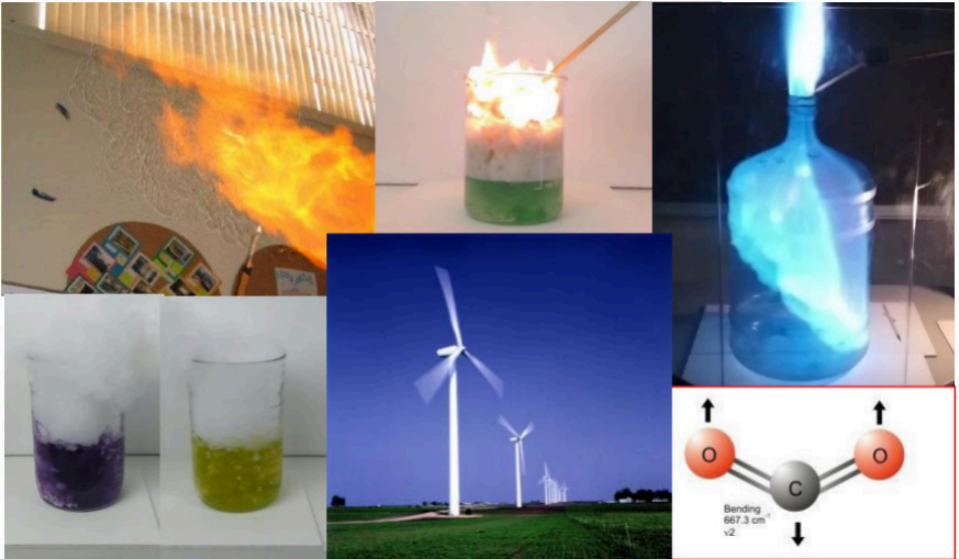

A Pollutant’s Tale and its primary school version, Gases in the Air, are two talks that have been developed and modified over the last ca. 18 years, that provide audiences from approximately 4–90 years old with the background to the composition of the Earth’s lower atmosphere, the Earth’s climate, and the impact of air pollution. In this article, we describe the content of the talks and provide videos of each experiment individually as well as a recorded performance of both talks to an empty auditorium. In this article, we discuss ways that the talk can be further developed and its impact on audiences.

Air pollution and climate change^1^ are two of the most pressing issues that the human race faces for the rest of this century. In 2005, the School of Chemistry at the University of Bristol established a Centre for Excellence in Teaching and Learning, called Bristol ChemLabS,^2−4^ that focused on innovating the way that practical chemistry was taught at higher education^5−7^ and in addition, developing a sustainable outreach program^2−4,8,9^ to school students, school teachers, the general public and a range of additional stakeholders, including businesses and Learned Societies. One of the first examples of outreach was the development of a talk called “A Pollutant’s Tale” that combined a journey through the Earth’s contemporary atmosphere (troposphere and stratosphere) combined with a wide range of “well-known” chemical experiments that were carried out to exemplify the contents of the talk.^10−14^ In addition to charting the impact of key pollutants such as VOCs (volatile organic compounds) and nitrogen oxides (in particular NO_2_), it also considers the impact of greenhouse gases such as CO_2_ and how important they are to maintaining a habitable average surface temperature for the Earth. The first time the talk was delivered was in 2006 (in the U.K.) and continues to be given by colleagues around the world. This version of the talk is given to audiences above the age of 12 typically (i.e., secondary school students in the U.K.). A version, without a supporting PowerPoint, focusing more on the Gases in the Air, was developed later (ca. 2008) and presented to primary schools (children aged 11 or younger in the U.K.). Both versions were developed by Prof. Dudley Shallcross and Tim Harrison, while Outreach Director and School Teacher Fellow of the Bristol ChemLabS CETL. Many variations of the original talks have been given, incorporating different experiments, and these are discussed later. We estimate that the two talks and their variants have been presented around 3000–4000 times, at schools, science festivals, interest groups, higher education institutes, and as part of teacher training in climate science and how to give a range of chemical demonstrations safely. In 2020, we recorded versions of both talks (in an empty lecture theater under the appropriate COVID-19 restrictions at the time), and links to these talks will be given later in this article. In addition, a wide range of “classic” chemistry demonstrations were recorded.^15^ It should be noted, of course, that these videos, while alerting the viewer to health and safety, take no responsibility for the safe conduct of any experiment and counsel the viewer to undertake a full risk assessment. In the remainder of this article, we describe the contents of the Pollutant’s Tale talk and provide links to video demonstrations of both talks, A Pollutant’s Tale and Gases in the Air. Any reader is welcome to use any or all of the PowerPoints (available from the corresponding author); a simple acknowledgment to the Bristol ChemLabS Outreach team is all that is required. As the Bristol ChemLabS Outreach program expanded, we began to work with colleagues from other countries and they helped to adapt the talk to incorporate cultural contexts; thus, several variations of the talk exist. In the next section, we describe the talk “A Pollutant’s Tale” and then discuss the merits of this format in the context of other climate education resources and provide a summary of previous evaluations of this project.

## A Pollutant’s Tale (APT) and Gases in the Air (GitA)

Both talks were recorded in 2020 and the links to these talks are

- **APT** with Chemistry experiment demonstrations^16^
- **APT** narrated powerpoint (ppt) only^17^
- **GitA** with Chemistry experiments (there is no accompanying powerpoint)^18^

The experiments described in “Gases in the Air” are a subset of those contained in a Pollutant’s Tale (**APT**) and so we refer readers to the YouTube recording and will concentrate on the description of **APT**.^15–18^

## Overview and Layout of APT

The talk begins by thinking about the three most abundant gases in the atmospheres of the planets in our solar system (not including Mercury) that are reproduced in Table 1 (note that the table used in the recorded talks is an earlier version of that presented in Table 1). This illustrates well that the composition of the Earth’s atmosphere is very different from our nearest neighbors (Venus and Mars) and different again from the giant gas planets. Of course, the reasons for the differences can be discussed with the audience, but we usually note that one major difference is the known existence of life on Earth. It then allows the opportunity to discuss hydrogen and helium, the major gases in the giant gas planets.^19^

**Table 1:** Three Most Abundant Gases in Each Planetary Atmosphere, Approximate Percentages^a^

name of planet	three most abundant gases
Jupiter	H_2_ (86.4%), He (13.6%), CH_4_ (0.02%)
Saturn	H_2_ (88%), He (12%), CH_4_ (0.05%)
Uranus	H_2_ (82.5%), He (15.2%), CH_4_ (2.3%)
Neptune	H_2_ (80%), He (19%), CH_4_ (1%–2%)
Venus	CO_2_ (96%), N_2_ (3.5%), SO_2_ (0.015%)
Mars	CO_2_ (95%), N_2_ (2.7%), Ar (1.6%)
Earth	N_2_ (78%), O_2_ (21%), Ar (0.93%)

a Data taken from ref (20).

## H_2_ and He

Balloons filled with each gas are then detonated, and the comparison between the two (flame and loud noise from detonating hydrogen compared with the ballon popping on detonation of helium) are discussed. Depending on the audience and time available, the depth of explanation of the differences in detonation are altered. For the post-16 school audiences, there may be a more involved discussion of the electronic structure and H and He and why H forms diatomic H_2_ gas while He is a monatomic gas at room temperature and atmospheric pressure for example. In early versions of the talk, we did include a pop test for H_2_ but found that there was not enough time in a 50–60 min presentation to include it. In recent times, this is an opportunity to discuss the potential use of hydrogen as a “clean” fuel, but up until ca. 2020, this was not discussed. Exploding a balloon containing each gas, is an excellent way to start the talk, one just popping (Helium), while the other providing an impressive flame and noise.^21,22^

## N_2_

The talk now focuses on the Earth’s atmosphere, we discuss why gaseous N forms N_2_, why it is a gas at room temperature and pressure, why it is effectively inert (lifetime of ca. 1 × 10^9^ years)^23^ and why an inert gas is important in the Earth’s atmosphere.^24^ If time permits, we discuss the importance of N being available to make certain proteins. However, for demonstration purposes, this gives an opportunity to show some experiments with liquid nitrogen (e.g., freezing flowers, eggs, rubber), which many of the audience will never have seen^25,26^ and can be connected back to the difficulties of making, storing, and handling liquid hydrogen in a potential hydrogen economy (i.e., liquid H_2_ is much colder than liquid N_2_, and we would have seen what exposing materials to liquid N_2_ does to them.^27−30^

## O_2_

Oxygen is the next most abundant gas in the Earth’s atmosphere, and we discuss the importance of photosynthesis to generate O_2_ in the Earth’s atmosphere.^31^ The Elephant’s Toothpaste experiment is well-known, for generating oxygen from the decomposition of hydrogen peroxide,^31^ it is dramatic and illustrates the impact of a catalyst as well to speed up a reaction (which we discuss later in the talk) and is a very useful demonstration for pre- and post-16 students.^32^

We did make oxygen from this reaction without adding washing up liquid to make the foam and could then relight a glowing spill to confirm that oxygen had been made,^33^ but again, this was dropped because of time. On some occasions, we have deliberately generated liquid oxygen using liquid nitrogen to condense it and demonstrated the magnetic properties of the blue liquid, but there is usually no additional time to complete this experiment.^34^ Depending on time and audience, we do include a short description of the production and importance of ozone in the stratosphere (the region between 10 and 50 km in altitude) from oxygen and may discuss natural catalytic loss.^35^

## Combustion of Fossil Fuels

Considering the impact of humans on air quality and climate change, we look next at the combustion of fossil fuels in the talk. First, illustrating incomplete combustion, depending on the audience, we talk about exothermic and endothermic reactions. The making and burning of acetylene (used as an early headlight) can be a very sooty reaction.^36^ Whereas the combustion of methanol in the “whoosh bottle” experiment produces a blue flame and illustrates complete combustion.^37^ The production of CO_2_ can be demonstrated from the residue (turns limewater milky, for example).

## NO_2_ as a Precursor to Tropospheric Ozone (O_3_)

An important pollutant from high-temperature combustion is nitrogen oxides, NOx (= NO + NO_2_). We show some urban air quality data from the U.K. (weekday) that shows morning and evening rush hour peaks of NOx and we then generate some NO_2_ gas, to demonstrate its brown color. We then describe how NO_2_ is rapidly dissociated by sunlight into NO and O atoms that add to the O_2_ molecules to form ozone, namely, O_3_. Not only is tropospheric ozone bad for air quality, but it is also a potent greenhouse gas and is responsible for accelerating lower atmosphere warming.^1^ The reaction used to make NO_2_ is an interesting one for senior school students, i.e., the reaction between copper turnings and concentrated nitric acid. Copper and dilute nitric acid do not react, but concentrated nitric acid and copper do react and form NO_2_, which we discuss.^38^

## CO_2_ (Dry Ice Experiments)

We then inspect a range of data that show that CO_2_ levels in the atmosphere have risen significantly in the last 200 years and that fossil fuel burning is the major factor, along with other human-based activity (we discuss ^13^C:^12^C ratios for CO_2_ and why this supports the assertion that the increase in CO_2_ arises from fossil fuel burning). We then carry out some experiments using dry ice (solid CO_2_), which shows how adding CO_2_ leads to water acidification, that solid CO_2_ sublimes directly to a gas (interesting chemistry) and fill a latex glove with a small amount and ask the audience to estimate by how much the glove will expand during the time left in the talk.^39−41^

## Mesosphere

In early versions of the talk, we did discuss the mesosphere (50–90 km in altitude) and the fact that when meteors ablate in this region of the atmosphere, they often release metals. This allows us to carry out some flame tests, but we did not have time to include this as the talk developed, and these experiments and demonstrations were rarely included after 2008. However, this can be included and linked to the Earth’s atmospheric structure.

As stated, the talk evolved with time (see Table 2 for a summary); the main change was the introduction of a discussion of stabilization wedges as a way to think about offsetting climate change (see next section). For APT, it is presented to a wide range of audiences in terms of age and background knowledge. Typically, for adult audiences, we spend more time on stabilization wedges and may explore all the options available and cut back on liquid nitrogen demos and dry ice experiments.

**Table 2:** Summary of Changes to APT with Time and Comments

original version (ca. 2006)	final version (ca. 2023)	comments
He balloon	He balloon	the ignition of He and H_2_ balloons are excellent starting demos for APT; in very recent versions, we extended the discussion of H_2_ ignition to discuss the emerging H_2_ economy, its climate offsetting but difficulty with generating and storing H_2_
H_2_ balloon	H_2_ balloon	
	brief discussion of the emerging H_2_ economy	
		
burning metal solutions (flame tests)	removed	no time to discuss the mesosphere and so no opportunity to discuss metals in this region
		
liquid nitrogen demos	liquid nitrogen demos	originally more time was given to liquid-nitrogen-based demos but new material on offsetting climate change was introduced instead.
freezing of rubber tubing, eggs, flowers, bananas	freezing of flowers or bananas	
shrinking of an air-filled balloon	shrinking of an air-filled balloon	
		
Elephant’s toothpaste	Elephant’s toothpaste	no change
		
methanol whoosh bottle	methanol whoosh bottle	reduction in combustion demos to just complete combustion to make more time for new material on offsetting climate change was introduced instead
acetylene production and ignition		
		
NO_2_ production	NO_2_ production	no change
		
CO_2_ experiments	CO_2_ experiments	no change
acidification	acidification	
sublimation	sublimation	
inflating of a plastic glove	inflating of a plastic glove	
		
	stabilization wedges and ways to offset climate change	a significant change to the original talk

### Stabilization Wedges

It soon emerged that we were not providing any hope for the future, and so we removed the experiments noted earlier to include a section on stabilization wedges. The concept, developed in 2005 by Pacala and Socolow,^42^ looks at ways to reduce carbon emissions over a period of 50 years and seeks to find seven “wedges”, where each wedge saves 1 billion tonnes of carbon emissions each year.^42^ Even back in 2005, there were 11 technologies considered that could offer a saving of 13 wedges of carbon, i.e., approximately double what was required. Some technology has improved significantly and is discussed in more detail elsewhere.^43^ Data from 2005 are summarized in Table 3. In a companion paper in this special edition, we discuss stabilization wedges in more detail. We have also adapted a simple model of the Earth’s climate^44^ that we sometimes incorporate into this talk and this too is the subject of another paper submitted to this special issue.

**Table 3:** Some Possible Ways to Offset Climate Change Using Technology Available in 2005^a^

technology	notes
improve fuel economy	increasing fuel economy from 30 mpg to 60 mpg for approximately 2 billion cars will save 2 wedges of carbon
reduce use of vehicles	decreasing miles driven for approximately 2 billion cars from 10,000 miles to 5,000 miles per year will save at least 1 wedge of carbon
more efficient buildings	reducing carbon emissions from heating buildings (e.g., better insulation) by 25% could save 1 wedge of carbon
improved power plant efficiency	assuming coal fired power plants are still in use by 2055, improving their energy generation efficiency from 40% to 60% would save 1 wedge of carbon
decarbonization of electricity and fuels	displacing 1400 GW of baseload coal with baseload natural gas would save 1 wedge of carbon
carbon capture and storage (CCS)	one wedge of carbon is saved by providing CCS at 800 GW of baseload coal plants or 1600 GW of natural gas plants
nuclear fission	a wedge of carbon can be saved by adding an additional 700 GW of energy from nuclear power
wind energy	using technology available in 2005, wind turbines produced about 50 GWp, increasing the capacity by a factor of 40 would save a wedge of carbon (in 2005 this equated to a wind farm covering a surface area similar to the size of Germany).
photovoltaic energy	in 2005, the total global deployment was 3 GWp, increasing to 2000 GWp by 2055 (factor of 700 increase) would save 1 wedge of carbon.
biofuels	in 2005, using first generation biofuels to save a wedge of carbon would require planting a crop covering 250 M hectares of land (approximately the size of India).
reducing deforestation	Planting new forests over an area the size of continental U.S. would save 1 wedge of carbon (based on 2005 data).

a Data taken from ref (42).

## Impact of the Talk

Several studies have been conducted concerning the impact of **APT** and **GitA** on a variety of stakeholders, and a summary is provided of the main findings.

### General Findings

Tuah et al.^13^ (and references therein) analyzed approximately 400 questionnaires collected up to 2009 from teachers and students (aged 11 and older) following a presentation of **APT** and reported several general observations. First, the demonstration experiments themselves are memorable and help the audience to remember key elements of the talk and the material covered.^45^ Second, the way that the presenter interacts with the audience, asking them to predict what might happen in an experiment or explain why something has happened helps to scaffold the learning.^46^ Third, orientating the audience (school) by knowing what they will have covered at that age in the school science syllabus^47,48^ was noted by teachers as being very effective in promoting understanding. Fourth, the talk was funny and entertaining, and the whole presentation was professional and took care to emphasize the importance of health and safety. Fifth, teachers noted that there was work involved for them to arrange a visit by the team or plan a trip to a venue, and so the event must be worth it. However, the Bristol ChemLabS’ outreach page contained a range of material on the content of the talk, health and safety information, and extended activities that could be undertaken back in school as well as curriculum links. Such pre- and post-demonstration supporting material helped the teachers to maximize the impact of the event.

### Gases in the Air^10^

Feedback from primary school teachers following a “Gases in the Air” presentation has been reported by Harrison et al.^10^ Key elements of the feedback include the following:

- The experiments are exciting, but the team ensures that the audience interacts and answers questions as they go along without losing focus.
- The language, terminology, and concepts described match current understanding extremely well.
- Misconceptions expressed through answers given by the audience are corrected in a gentle way, and nonscientific phrases are addressed by offering alternatives.
- The length of the talk (ca. 30 min) is ideal for this wide age group.

The event was very funny, and it was great to see that children could associate fun with science. The teachers learned new material. A general concern with primary schools^10^ was giving a presentation that would excite the children and raise a lot of questions that the children would ask their teacher in the days or weeks after that the teacher could not answer. This would be a problem for these teachers, as they would not know whether the question was sensible or not or how to answer those that were sensible. First, in collaboration with U.K. award winning primary school science teachers^49^ a previsit document was prepared that teachers could read and prepare themselves for the visit. In later years online sessions with the teachers were offered before visiting. We also employed a few different methods to help answer subsequent questions, the first being that teachers recorded the questions and sent them in bundles to us, and we answered them and sent them back. This was very helpful to us, as we were able to modify the talk to answer frequent questions while engaging with the children. Second, we began to run online sessions with the school and answer questions that the children had. There was less need to do this for secondary school students as their teachers were more able to answer chemistry-specific questions, but some more climate-related questions required help, either by answering an email from the teacher or sometimes by follow-up sessions. Primary school children’s questions focus on climate change with some regular examples listed.

- What happens if the Earth gets too hot/too cold?
- How much longer could the planet survive if we do not change?
- What would happen if all the ice melted because of global warming?
- What are you most afraid of about climate change?
- In your opinion, are we taking serious action about climate change?
- Out of all the things we can do to help, which is the most effective?
- Are any other planets impacted by climate change?

As we noted in the description of the demonstrations, we adapted the presentation over time. The most significant change was the introduction of a climate action section and the concept of stabilization wedges to offset carbon emissions. This addition meant removing flame tests and reducing the number of other demonstrations (e.g., combustion of acetylene, the number of liquid nitrogen experiments for example). Most recently, with the growing interest in the hydrogen economy, we have made more reference to this and noted the challenges to storing and transporting liquid hydrogen. As teachers and the public provide feedback on the presentations, we have made minor alterations to comments made, and when presenting in different countries, we would be sensitive to issues that may be relevant there and not in the U.K.

It has been noted that the success of the two talks (as demonstrated by positive feedback, numerous rebookings and being incorporated into outreach activities in other UK HEIs and colleagues abroad)^10−13^ is the combination of exciting and memorable experiments, appropriately pitched talks that explain and engage with the audience and a strong sense of fun in a safe environment. Combining the expertise of a very experienced secondary school chemistry (science) teacher and an academic in HE who specialized in atmospheric chemistry allowed the initial presentations to be well-received. The strong story-telling basis^50,51^ of the talk was also noted by many to aid comprehension.^10−12^ All presenters were trained for some time (typically months) before they participated in presenting talks and became lead presenters only after ca. 6 months. In this way, the talk did not suffer from deviating significantly from the original script with the potential for erroneous commentary to slip into the talk, and of course, the lead presenter was able to set up and carry out all experiments safely. It should also be noted that giving such presentations incurs costs, financially in terms of person time, travel, and subsistence costs, and non-negligible costs for consumables given the frequency of presentation. In addition, regular access to liquid nitrogen, dry ice, and high concentration hydrogen peroxide is difficult unless part of a HE Chemistry department. When presenting overseas, we worked with partner chemistry departments to provide chemicals. In a future paper, we will discuss sustainable outreach in more detail, but academic research grants supported this activity over the majority of the period by 50% or more. The talk was versatile enough that particular research projects on atmospheric chemistry could be incorporated into the talk and provide a method where that research could be shared with live audiences. Sponsorship from learned societies and companies was also able to support this activity, and some recipients were able to make a financial contribution to the presentation. In this way, the large number of presentations did not erode the School of Chemistry funds at Bristol University and indeed provided many additional benefits (e.g., a REF Case study in 2014 and 2020 in the U.K.).

The studies^10−13^ noted the impact on other presenters, often post-graduate students (Ph.D.s),^8^ but also undergraduate students in South Africa in particular.^11,12,52^ These presentations developed a range of important transferrable skills, planning the activity, and ensuring that all materials are packed safely and available for set up on arrival at the venue. Logistics in terms of knowing where the venue was and the best route there, was there parking? Are all important planning skills. Ensuring that the venue and setup are safe, starting and finishing on time, and delivering the presentation safely. All presenters remarked that the first few times were difficult, but the buzz from a successful delivery and the feedback from the audience was a significant confidence builder. As we have discussed in a previous paper,^8^ students often reported that they were able to draw from this experience to demonstrate key skills in job interviews and were themselves much more confident. Although drawn together initially through public engagement, many research (chemistry and chemistry education)^53−56^ studies were generated through this initial collaboration. The reflective cycle is central to the continued development of this presentation, taking on board feedback allows the team to modify over time aspects of the talk to improve clarity. This has meant, as noted, that certain experiments have been removed in order to make time to allow either an explanation or the insertion of new material. It is also noteworthy that teachers have also porvided feedback that they learned new material and welcomed the opportunity to have some CPD.^57^ Extensive feedback over the whole period showed that audiences were better informed about climate change and air pollution as a result of attending, and those that viewed the talk before and after the introduction of the section on stabilization wedges welcomed this addition.

As noted, it has been possible to obtain feedback from the audiences attending the talk; it is much harder to provide evidence of empowering impact or actions in the long term. However, the research by Sewry and Paphitis,^52^ which provides detailed analysis of the implementation and impact of APT, shows long-term impact on undergraduate students giving the talks and the school students who were the audience. There are many instances of feedback that suggest a deeper level of engagement and of schools embarking on projects that address and assess their climate impact.

Since **APT** was devised, a number of online resources on climate education have been developed and are very useful. However, the effectiveness of live demos mixed with multimedia is still very effective after nearly 20 years. The interactive nature of the talk, where the presenters “scaffold” the learning, as suggested by Vygotsky,^58^ aids interaction and learning. Many researchers have reported increased levels of task involvement,^59^ participation,^59,60^ curiosity,^60^, and engagement,^61^ following a lecture demonstration, and that information is retained and processed for some time afterward.^62^ The response of the crowd to dramatic live demonstrations allows these interactions to be highly memorable, as we have seen in science festivals, for example, where audiences can interact with presenters after the event. Using demonstrations that re-emphasize concepts that the audience is likely to already know is a good way to demystify climate and air pollution. Climate change education is becoming more prominent in school curricula, and talks such as **APT** are a good way to either introduce or summarize the topic.

## Summary and Conclusion

“The Pollutant’s Tale” and its primary school variant, “Gases in the Air”, have been presented on numerous occasions over an 18 year period. The talk uses many well-known chemistry demonstration experiments to support the discussion of air pollution and climate change for a wide variety of audiences. The talk has benefitted from strong input from school teachers, who have shaped the language used and science concepts discussed so that it is as appropriate as possible for a given audience. Development of a significant core team of presenters has ensured that it has been available to a wide number of audiences, and in so doing, a myriad of benefits have accrued for those presenters, particularly undergraduates and post-graduates. Funding is an important consideration, and we have been able to diversify sources of funding so that it can be either free of charge to schools or at a very reduced cost. There is strong evidence that the content of the talk is understood by these wide-ranging audiences and that this is a good vehicle to disseminate the elements of the Earth’s climate.
